# Use of shock index as a prognostic marker in patients with normal heart rate and blood pressure at ICU admission

**DOI:** 10.1186/2197-425X-3-S1-A596

**Published:** 2015-10-01

**Authors:** FG Zampieri, F Colombari

**Affiliations:** Intensive Care Unit, Hospital Alemão Oswaldo Cruz, São Paulo, Brazil; Intensive Care Unit, Hospital das Clínicas, University of São Paulo, São Paulo, Brazil

## Introduction

Heart rate (HR) and blood pressure (BP) are two of the most ubiquitous hemodynamic parameters assessed in medicine. Nevertheless, normal values of HR and BP are not absolute markers of stability. Shock index (SI - ratio between HR and systolic BP) is an important prognosis marker in trauma [1] and has been suggested to be associated with mortality in acutely ill patients even when HR and BP are within the normal range [2]. Intriguingly, there is a lack of data reporting SI as a marker of severity in the intensive care unit. It is especially unclear if SI could be an additional useful parameter in patients with normal values of HR and BP.

## Objectives

To assess the association between SI measured at admission and mortality in otherwise stable patients admitted to the ICU. As secondary endpoint, we assessed the association between SI and subsequent need for organ support (vasopressors, mechanical ventilation and renal replacement therapy).

## Methods

Retrospective analysis of all patients admitted from January 2012 until August 2014 in a tertiary ICU in Brazil. Inclusion criteria assessed at ICU admission were:

1. HR > 50 and < 100 bpm,
2. Systolic blood pressure > 90 mmHg,
3. Mean BP > 65 mmHg,
4. Absence of vasopressor use.

Association between SI and hospital mortality and need for organ support was assessed through logistic regression controlling for illness severity (SAPS3 without attributable points for HR and BP).

## Results

**Figure 1:**
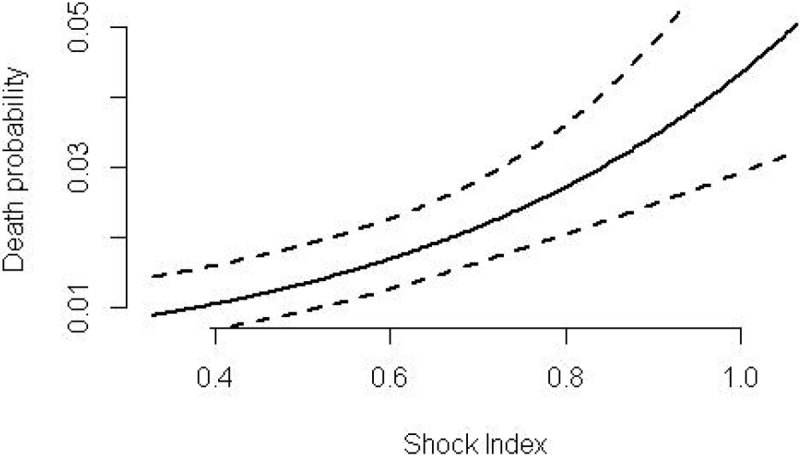
Figure 1

3,140 patients were included. Hospital mortality was 5.7% (179 patients). 7.3% (231 patients) eventually required vasopressors; 4.3% (137) required mechanical ventilation and 2.6% (83) required renal replacement therapy. Higher SI was independently associated with mortality (Figure 1)

An SI greater than 0.7 had an odds ratio for mortality of 1.43 (95% CI 1.03-2.00; p = 0.03). A SI value higher than 0.7 was associated with need for starting vasopressors during ICU stay (OR 1.69; 95% CI 1.28-2.23; p < 0.01). SI was not associated with need for mechanical ventilation or renal replacement therapy. Excluding patients with a known diagnosis of chronic heart failure did not change the results.

## Conclusion

In a population with otherwise stable vital signs at admission, higher SI values were independently associated with mortality and need for vasopressors. SI should be considered as an additional prognostic marker in this population and may help predicting future hemodynamic instability.
